# A Soft-Threshold Filtering Approach for Tomography Reconstruction from a Limited Number of Projections with Bilateral Edge Preservation

**DOI:** 10.3390/s19102346

**Published:** 2019-05-21

**Authors:** Tiago T. Wirtti, Evandro O. T. Salles

**Affiliations:** Programa de Pós-graduação em Engenharia Elétrica (PPGEE), Departamento de Engenharia Elétrica, Universidade Federal do Espírito Santo, Campus Goiabeiras. Av. Fernando Ferrari, 514, Goiabeiras, Vitória-ES 29075-910, Brazil; ppgee@ele.ufes.br

**Keywords:** signal processing, tomography, image reconstruction, X-ray imaging, Bayesian statistics

## Abstract

In X-ray tomography image reconstruction, one of the most successful approaches involves a statistical approach with $l_{2}$ norm for fidelity function and some regularization function with $l_{p}$ norm, 1<p<2. Among them stands out, both for its results and the computational performance, a technique that involves the alternating minimization of an objective function with $l_{2}$ norm for fidelity and a regularization term that uses discrete gradient transform (DGT) sparse transformation minimized by total variation (TV). This work proposes an improvement to the reconstruction process by adding a bilateral edge-preserving (BEP) regularization term to the objective function. BEP is a noise reduction method and has the purpose of adaptively eliminating noise in the initial phase of reconstruction. The addition of BEP improves optimization of the fidelity term and, as a consequence, improves the result of DGT minimization by total variation. For reconstructions with a limited number of projections (low-dose reconstruction), the proposed method can achieve higher peak signal-to-noise ratio (PSNR) and structural similarity index measurement (SSIM) results because it can better control the noise in the initial processing phase.

## 1. Introduction

X-ray computer tomography (CT) measures the attenuation of X-ray beams passing through an object, generating projections. Such projections are processed, resulting in an image (slice) of the examined object. This is known as a CT image reconstruction. The CT scan, formed by concatenating a large number of adjacent reconstructed images, has been proven to have great value in delivering rapid and accurate diagnoses for many cases in modern medicine. Although CT scanning has evolved considerably since its creation in 1972 by Godfrey Hounsfield, only recently has the concern with radiation levels in radiological examinations become important, leading to the “as-low-as-reasonably-achievable principle”, known as the ALARA principle. The ALARA principle states that only the minimum amount of radiation must be applied to the patient. For this reason, ALARA is widely accepted in the medical CT community [1]. To reduce the X-ray dose of the patient during the CT scan, there are two possibilities: (1) reduce the amount of projection (the quantity of X-rays emitted) during the CT scan or (2) reduce the power of the X-ray source during image acquisition. Both cases generally lead to low-quality reconstructed CT images. Then, a state-of-the-art problem is to propose a method that allows good-quality CT image reconstruction with a low-dose X-ray source. The central theme of this work is the reconstruction of images from the signal of the CT process, where the X-ray dosage is a concern. Before discussing CT image reconstruction approaches with low-dose X-rays, the next sections will present the most important classical, iterative, and statistical techniques to enable the reader to understand how CT image reconstruction has evolved to the current state of the art.

### 1.1. Classical, Iterative, and Statistical CT Image Reconstruction Approaches

The first approach to become popular, especially for its performance, was the filtered backprojection (FBP) reconstruction technique [2,3]. FBP is a classic method based on the Fourier central slice theorem and is implemented with the fast Fourier transform (FFT). Although it exhibits good performance, FBP has difficulty in being adapted to new CT scanner architectures. FBP requires high-dose radiation (in comparison to modern methods) and is not consistent with the ALARA (as-low-as-reasonably-achievable) principle [1].

The classical approaches, while successful, do not favor the incorporation of physical-statistical phenomena in the CT framework. For example, photon emission is a rare event and may be well described by the Poisson distribution [4]; beam behavior is best described by a response function that models the shadows cast onto detectors using a Gaussian model [5]; the beam hardening phenomenon (lines and shadows adjacent to high-density reconstructed areas) that appears due to the polyenergetic nature of X-ray emissions can be statistically corrected [6]; the loss of photons by sensors, known as photon read-out, is a Gaussian phenomenon [7]; data acquisition electronic noise and energy-dependent signals can be modeled as compound Poisson plus Gaussian noise [8]; etc. In this context, a statistical approach means adding to the mathematical model elements that describe physical-statistical phenomena present in the CT image reconstruction process. Therefore, the incorporation of detailed statistical models into CT reconstruction is not straightforward. In this sense, many solutions for the CT image reconstruction problem use some form of statistical approach [9,10,11,12,13,14,15,16,17,18,19,20]. Adaptive statistical iterative reconstruction techniques have shown significant results compared to non-adaptive techniques [17,18,21,22]. In general, although the models incorporate part of the statistical phenomena, most of these phenomena are not modeled since the practical effects are relatively insignificant and/or result in high-cost computational solutions.

An important method is proposed by Clark et al. [23], which consists of using rank-sparse kernel regression filtration with bilateral total variation (BTV) to map the reconstructed image into spectral and temporal contrast images. In this work, the authors strictly constrain the regularization problem while separating temporal and spectral dimensions, resulting in a highly compressed representation and enabling substantial undersampling of acquired signals. The method (5D CT data acquisition and reconstruction protocol) efficiently exploits the rank-sparse nature of spectral and temporal CT data to provide high-fidelity reconstruction results without increased radiation dose or sampling time. However, a remark should be made regarding the use of BTV (regularization based on $l_{1}$ norm). This often leads to the piecewise constant result and hence tends to produce artificial edges on the smooth areas. In order to mitigate this counterpoint of $l_{1}$ norm regularization, Charbonnier et al. [24] developed an edge-preserving regularization scheme known as bilateral edge preservation (BEP), which allows the used of an $l_{p}$ norm, 1<p<2, and is applied in this work. Sreehari et al. [25] proposed a plug-and-play (P&P) priors framework with a maximum a posteriori (MAP) estimate approach used to design an algorithm for electron tomographic reconstruction and sparse image interpolation that exploits the non-local redundancy in images. The power of the P&P approach is that it allows a wide array of modern denoising algorithms to be used as a prior model for tomography and image interpolation. Pirelli et al. [26] propose the denoising CT generalized approximate message passing algorithm (DCT-GAMP), an adaptation of approximate message passing (AMP) techniques that represents the state of the art for solving undersampling compressed sensing problems with random linear measurements. In contrast, this approach uses minimum mean square error (MMSE) instead of MAP, and the authors show that using MMSE favors decoupling between the noise conditioning effects and the system models.

The Bayesian statistical approach is widely applied to the reconstruction of X-ray tomographies, with some variations, [14,15,16,18,27,28], and makes it possible to insert prior knowledge into the CT system model. This approach promises two advantages. First, it provides the search with more satisfactory solutions (noiseless ones) through the limitation of the searchable set of solutions using an a priori function (known as restriction). This restriction may mean that, for example, very high variations (high frequencies) between neighboring pixels will be considered as noise (and therefore must be discarded), and moderate frequencies will be considered as edges (and therefore must be preserved). In the context of computed tomography, this means that large differences in intensity between neighboring pixels tend to be interpreted as noise, and therefore, such a solution should be disregarded [5]. Moreover, solving the CT reconstruction system, y=Ax+e, is an inverse and ill-posed problem, and prior knowledge often ensures the stability of the solution. Second, we can adopt a simplified mathematical model for tomographic image reconstruction and compensate its inefficiency (instability, noisy reconstruction, etc.) by adding the statistical component (prior knowledge) to the objective function. However, the model simplification has its limitations and should be used restrictively [5]. As a consequence of prior knowledge introduction, more satisfactory solutions (low noise level) can be found. Maximum a posteriori (MAP) is a useful statistical framework for CT reconstruction [12,15,16,27,28] and favors the incorporation of the regularization term with prior knowledge into the model. The MAP strategy provides an objective function composed by the sum of the probability (also known as fidelity) and the regularization function that establishes the optimization restriction criteria, also known as a prior.

### 1.2. Signal Modeling and Error Considerations in CT Image Reconstruction

**Figure 1:**
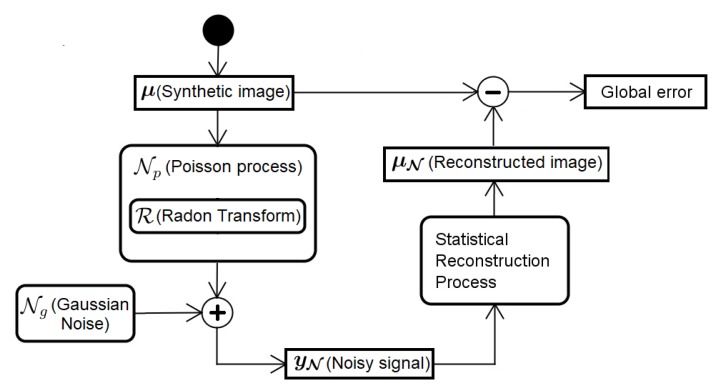
From acquisition to reconstruction and measurement of error.

As previously discussed, the statistical approaches can reduce deficiencies caused by classical mathematical modeling without having to literally incorporate the complexity of a real-world model. However, before proposing a statistical (non-deterministic) model that results in a lower noise reconstruction, it is necessary to establish the process as a whole. As shown in Figure 1, the process begins with a synthetic image, μ.

In this work, we use different synthetic images, namely Shepp–Logan head phantom and FORBILD head and abdomen phantom definitions [2,29]. The synthetic image is submitted to the Radon transform, $R\left(.\right)$, generating the ideal (free of noise) signal of the CT scan. The acquisition of data in the CT equipment depends on the amount of photons that reaches each detector. Once this problem is a particle countable process, well described by Poisson statistics [8], it is used in the model in Figure 1, represented by the $N_{p}$ random variable. However, due to the high number of photons, the acquisition process can be modeled as Gaussian due to the central limit theorem. In addition, the Gaussian model leads to additive algorithms, whereas the Poisson model leads to multiplicative, and therefore less efficient, algorithms [24]. In this work, we assume the signal arriving at the CT equipment detectors is influenced by Gaussian additive noise and, from among the dosage reduction methods presented in Section 1, we chose to emulate the low radiation dosage by reducing the number of projection angles processed. This means we assume that each detector absorbs an amount of photons that allows modeling the noise as a Gaussian additive, and the low dosage occurs by reducing the number of projections captured by the detectors (by reducing scanning angles). Accordingly, even with the process having a Poissonian nature, Gaussian additive noise can be added to the process, as

(1) $$y_{N}=R\left(\mu \right)+N_{g},$$

where $y_{N}$ is the resulting signal that approximates a tomography signal, $R\left(\mu \right)$ is the result of applying the Radon transform on the synthetic image, μ, and $N_{g}$ is the Gaussian additive noise.

The remainder of this work is dedicated to the reconstruction of the CT image, $\mu_{N}$, from the signal, $y_{N}$, and the reduction of the global error, i.e., the reduction of the difference between synthetic and reconstructed images. As a criterion for measuring the quality of image reconstruction, we use peak signal-to-noise ratio (PSNR) and structural similarity, known as SSIM [30].

### 1.3. The Contribution of This Work

This work proposes a MAP solution with adaptive regularization term modeled by a bilateral edge-preserving (BEP) function [31,32] to regularize the fidelity term ($l_{2}$ norm). The result of BEP regularization is then subject to regulation by the total variation (TV) of the discrete gradient transform (DGT) function. The results of reconstruction with an adaptive norm, $l_{a}$, using BEP are compared via structural similarity (SSIM) and peak signal-to-noise ratio (PSNR) with a simultaneous algebraic reconstruction technique (SART) reconstruction regularized via the TV minimization of the discrete gradient transform (DGT) function. The image reconstruction is an iterative process, and SSIM is calculated for each step, making it possible to objectively compare the methods step by step. The assumption is that the better the reconstruction method, the higher the SSIM value associated with the reconstructed image. We also use the well-known PSNR metric to compare the process of image reconstruction step by step. The proposal is to determine if both SSIM and PSNR present consistent results in comparison to each other. Both approaches use synthetic images (the same used to generate the input signal, $y_{N}$) as a reference (for comparison). The rest of this work is organized as follows. In Section 2, the MAP model is developed, resulting in the objective function. In Section 3, the optimization technique of the objective function is developed. In Section 4, experiments are performed, and the results are presented and analyzed. Finally, in Section 5, we present the conclusions and final considerations.

## 2. Modeling the Objective Function

Most modern CT scanners use energy integration detectors whose photon counts are proportional to the total energy incident on them. Energy, in turn, is proportional to the number of X-ray photons that affect the detectors (sensors) of the tomograph. The denser the region traversed by X-ray photons, the lower the count $I_{i}$ of detected photons over integral line $L_{i}$, $i=1,...,N_{I}$, where $N_{I}$ is the maximum number of projections acquired by the CT scanner. This is known as the Beer–Lambert law, defined as

(2) $$I_{i}=I_{0}exp\left(-\int_{L_{i}}\mu \left(x,y\right)dL\right),i=1,...,N_{I},$$

where $I_{0}$ is the number of detected photons when the beam finds no obstacle, and the exponential term is the integral of all linear attenuation coefficients $\mu \left(x,y\right)$ on the line $L_{i}$ (with $\left(x,y\right)$ being 2-D coordinates following $L_{i}$), which is the path of the beam. Equation (2) assumes that every X-ray emission has the same energy level, meaning that the process is monoenergetic. This approach is adopted in many works such as [9,10,11,15,16,27,28,33,34,35], with the advantage of avoiding the beam hardening problem. Moreover, the monoenergetic approach leads to a more tractable mathematical model. However, the emission of X-rays is, by nature, polyenergetic. As a consequence, the same object reacts differently when subjected to X-rays of different energy levels, generating unwanted artifacts in the reconstructed image. These defects can be avoided, but with the adoption of complex models as in [12,13,36] and at a high computational cost. This topic is complex and still subject to change because CT scanners using monoenergetic X-ray sources are beginning to emerge [37].

In favor of a better understanding of the purpose of this work, we first present a base solution that uses SART reconstruction regularized via TV minimization of the DGT function (SART+DGT), highlighting the relevant parts, and then we present our approach. This strategy is trustworthy because makes it clear the value of the contribution in this work. The proposed method is abbreviated as SART+BEP+DGT.

### 2.1. Objective Function Modeling Using Soft-threshold Filtering for CT Image Reconstruction

This method consists of modeling an objective function with a $l_{2}$ norm fidelity function added and a DGT prior function regularized by a $l_{1}$ norm with TV minimization. Optimization of the objective function (Section 3) is performed using alternating minimization. The fidelity term is minimized by SART. The regularization (prior minimization) is performed by constructing a pseudo-inverse of the DGT and adapting a soft-threshold filtering algorithm whose convergence and efficiency have been theoretically proven by [38].

The key aspect of the modeling process is that reconstruction estimates the discrete attenuation, $\mu \left(x,y\right)$ for each *j* pixel of the image, with $j=1,\ldots ,N_{J}$. Thus, the integral over the line, $p_{i}=\int_{L_{i}}\mu \left(x,y\right)dL$, can be discretized as

(3) $$p_{i}\approx \sum_{j=1}^{N_{J}}a_{ij}\mu_{j}={\left[A\mu \right]}_{i},i=1,\ldots ,N_{I},$$

where $A={\left\{a_{ij}\right\}}_{N_{I}\times N_{J}}$ is the matrix representing the system geometry, $\mu ={\left(\mu_{1},\ldots ,\mu_{N_{J}}\right)}^{T}$ is the linear attenuation coefficient vector with $\mu_{j}$ representing the *j*-th pixel, and the symbol *T* is the transpose of the matrix. In this model, every $a_{ij}$ is defined as the normalized length of the intersection between the *i*-th projection beam and *j*-th rectangular pixel centered in $\left(x,y\right)$. The emission of X-ray photons is a rare event, so a Poisson distribution is usually adopted to describe the probabilistic model, expressed as

(4) $$y_{i}∼Poisson\left\{{\bar{y}}_{i}=I_{0}e^{-p_{i}}\right\},i=1,\ldots ,N_{I},$$

where $y_{i}$ is the projection (measurement) along the *i*-th X-ray beam, and $\bar{y_{i}}$ is the expected value. Because the X-ray beams are independent from each other, taking into account Equation (4), the joint probability of $y=\left\{y_{1},y_{2},...,y_{N_{I}}\right\}$ given μ, $P\left(y|\mu \right)$ and observing $y_{i}$ countable events may be expressed as

(5) $$P\left(y|\mu \right)=\prod_{i=1}^{N_{I}}P\left(y_{i}|\mu \right)=\prod_{i=1}^{N_{I}}\left(\frac{{\left({\bar{y}}_{i}\right)}^{y_{i}}}{y_{i}!}e^{-{\bar{y}}_{i}}\right).$$

Using the MAP approach, as in [9,15,16,27], we have the objective function as follows:

(6) $$\Phi \left(\mu \right)=\sum_{i=1}^{N_{I}}\frac{y_{i}}{2}{\left({\left[A\mu \right]}_{i}-{\hat{p}}_{i}\right)}^{2}+R_{DGT}\left(\mu \right),$$

where $\sum_{i=1}^{N_{I}}\frac{y_{i}}{2}{\left({\left[A\mu \right]}_{i}-{\hat{p}}_{i}\right)}^{2}$ is the fidelity term, with ${\hat{p}}_{i}$ being an estimate of $p_{i}$, and $R_{DGT}\left(\mu \right)$ (multiplied by a factor β=1 ignored in Equation (6)) is the regularization term with $l_{1}$ norm based on DGT, defined as

(7) $$D_{j}\mu =D_{m,n}\mu =\sqrt{{\left(\mu_{m,n}-\mu_{m+1,n}\right)}^{2}+{\left(\mu_{m,n}-\mu_{m,n+1}\right)}^{2}},$$

where $j=\left(m-1\right)\times W+n$, m=1,2,…,H, n=1,2,…,W, with *W* and *H* being, respectively, the width and height of the matrix representing the image with $N_{J}=W\times H$ pixels. By definition, TV is the sum of DGT for all pixels of the image:

(8) $$TV\left(\mu \right)={∥D\mu ∥}_{1}=R_{DGT}\left(\mu \right),$$

with $D\mu ={\left(D_{1}\mu ,\ldots ,D_{NJ}\mu \right)}^{T}$. Thus, introducing the auxiliary variable ν=Dμ and applying the transformation

(9) $$A_{\Lambda }=\Lambda A=\left\{a_{\Lambda_{i}j}\right\},{\hat{p}}_{\Lambda }=\Lambda \hat{p},\hat{p}=\left({\hat{p}}_{1},{\hat{p}}_{2},...,{\hat{p}}_{NI}\right),$$

with $\Lambda =diag\left(\sqrt{y_{i}/2}\right)\in R^{N_{I}}\times R^{N_{I}}$ being a diagonal matrix, the objective function in Equation (6) can be rewritten as

(10) $$\Phi \left(\mu \right)=∥A_{\Lambda }\mu -\hat{p_{\Lambda }}{∥}_{2}^{2}+\beta {∥\nu ∥}_{1},\nu =D\mu ,$$

where β is a positive adjustment parameter to balance the terms of fidelity and TV and is usually set to 1 [12,16]. The ultimate goal is to minimize the objective function $\Phi \left(\mu \right)$, obtaining $\hat{\mu }$, as shown below:

(11) $$\begin{array}{c} \hat{\mu }=\underset{\mu }{argmin}\left\{F\left(\mu \right)-\beta R\left(\mu \right)\right\}, \end{array}$$

where the fidelity term, $F\left(\mu \right)$, represented both in the expanded version, as in Equation (6), and in the compact version, as in Equation (10), is shown below as

(12) $$F\left(\mu \right)=\sum_{i=1}^{N_{I}}\frac{y_{i}}{2}{\left({\left[A\mu \right]}_{i}-{\hat{p}}_{i}\right)}^{2}={∥A_{\Lambda }\mu -{\hat{p}}_{\Lambda }∥}_{2}^{2},$$

and $R\left(\mu \right)$ is the restriction that drives the solution according to certain criteria ($l_{1}$ norm in this case). The optimization of $F\left(\mu \right)$, although simple, is an important concept and can be defined as follows:

(13) $$\tilde{\mu }=\underset{\mu }{argmin}\left\{F\left(\mu \right)\right\}.$$

Inspired by the model in Equation (10), we propose in what follows a method for CT image reconstruction using adaptive soft-threshold filtering, which means, in brief, that the proposed method is intended to balance edge preservation and noise mitigation.

### 2.2. Objective Function Modeling by Using Bilateral Edge Preservation for CT Image Reconstruction

Regularization using the DGT ($l_{1}$ norm) works well for the CT reconstruction problem because it searches among the solutions of fidelity ($l_{2}$ norm) optimization looking for the one with a lower TV. However, it is common sense that the regularization based on the $l_{1}$ norm often introduces artificial edges in smooth transition areas. Moreover, a good regularization strategy must simultaneously perform noise suppression and edge preservation. With this motivation, Charbonnier et al. [24] proposed the bilateral edge preserving (BEP) regularization function, inspired by the bilateral total variation (BTV) regularization technique [39]. BTV regularization is defined by

(14) $$R_{BTV}\left(X\right)=\underset{l+m\geq 0}{\underset{︸}{\sum_{l=-q}^{q}\sum_{m=0}^{q}}}\alpha^{\left|l|+|m\right|}{∥X-S_{x}^{l}S_{y}^{m}X∥}_{1},$$

where *q* is a positive number, $S_{x}^{l}$ and $S_{y}^{m}$ are displacements by *l* and *m* pixels in the horizontal and vertical directions, respectively, X is the image in reconstruction/regularization, and α, 0<α<1, is applied to create a spatial decay effect for the sum of terms in BTV regularization. The BEP regulation uses the same principle as BTV but with an adaptive norm (instead of the $l_{1}$ norm) defined by

(15) $$\rho \left(s,a\right)=a\sqrt{a^{2}+s^{2}}-a^{2},$$

where *a* is a positive value and *s* is the difference that one wants to minimize. This function was initially proposed by [24] to preserve edges in the image regularization process. The parameter *a* is used to specify the error value for which the regularization becomes linear (growing with the error) to constant (saturated, regardless of the error). The same adaptive norm definition is also used in super-resolution problems [32]. The $\rho \left(s,a\right)$ function is an M-estimator since it corresponds to the maximum likelihood (LM) type estimation [40], and has its influence function given by

(16) $$\psi \left(s,a\right)=\frac{\partial \rho \left(s,a\right)}{\partial s}=\frac{as}{\sqrt{a^{2}+s^{2}}}.$$

**Figure 2:**
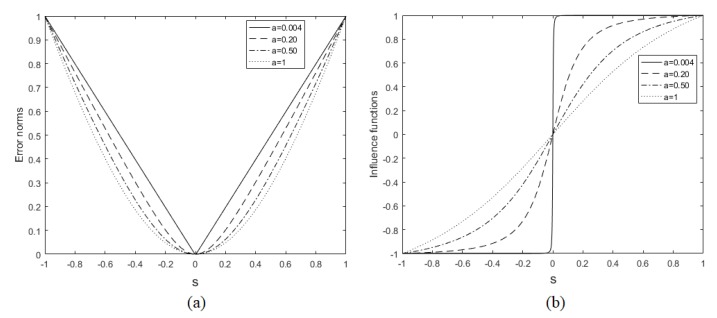
(**a**) Error norm function, Equation (15), and (**b**) influence function, Equation (16).

The influence function indicates how much a particular measure contributes to the solution [32]. We illustrate in the graphs of Figure 2a the behavior of $\rho \left(s,a\right)$ (the error norm function), and in Figure 2b, its influence function. It can be observed that as parameter *a* evolves from 0 to 1, the function changes its behavior from $l_{1}$ to $l_{2}$ norm. Thus, as mentioned by [31] and [32], Equation (15) behaves adaptively with respect to the norm that it implements.

Therefore, combining Equations (14) and (15) for the particular case of tomography reconstruction, we propose an adaptive operator defined as

(17) $$R_{BEP}\left(\tilde{\mu }\right)=\underset{l+m\geq 0}{\underset{︸}{\sum_{l=-q}^{q}\sum_{m=0}^{q}}}\sum_{j=1}^{N_{j}}\alpha^{\left|l|+|m\right|}\rho_{a}\left({\tilde{\mu }}_{j}-S_{x}^{l}S_{y}^{m}{\tilde{\mu }}_{j}\right),$$

where *q*, α, $S_{x}^{l}$, and $S_{y}^{m}$ are the same as in Equation (14); $\tilde{\mu }$, defined in Equation (13), is the estimated image obtained in the *i*-th iteration by $l_{2}$ minimization of the objective function in Equation (12); and ${\tilde{\mu }}_{j}$ is the *j*-th pixel of image $\tilde{\mu }$, with $j=1,\ldots ,N_{j}$. It is noteworthy that the term $R_{BEP}\left(\tilde{\mu }\right)$ imposes an $l_{a}$ regularization norm, 1<a<2, on the image $\tilde{\mu }$. Thus, we can rewrite the objective function of Equation (10), $\Phi \left(\mu \right)$, so that a new regularization term, $R_{BEP}\left(\tilde{\mu }\right)$, is introduced between the $l_{2}$ norm minimization and TV minimization. As a consequence, the objective function proposed in this paper incorporates adaptive regularization to the objective function, and defining an auxiliary variable $\sigma =R_{BEP}\left(\tilde{\mu }\right)$, we have

(18) $$\Phi \left(\mu \right)=∥A_{\Lambda }\mu -\hat{p_{\Lambda }}{∥}_{2}^{2}+{\gamma ∥\sigma ∥}_{\eta }+\beta {∥\nu ∥}_{1},$$

where γ is a positive adjustment parameter to balance the terms of fidelity and adaptive regularization. The other parameters are the same as in Equation (10), and η, 1≤η≤2, is the norm BEP method imposed on the regularization process.

## 3. Objective Function Optimization

The alternating minimization technique makes the simultaneous optimization of two or more terms of an objective function possible. Thus, for the proposed method, three steps are necessary: (1) minimizing $F\left(\mu \right)$ with SART, (2) applying the gradient descent (GD) method to the result of the first stage, using $R_{BEP}\left(\mu \right)=\gamma {∥\sigma ∥}_{\eta }$ as a regularization term, and (3) applying DGT regularization to the previous result, minimizing ${\beta ∥\nu ∥}_{1}$ with soft-threshold filtration. The three stages are repeated iteratively until a satisfactory result is obtained or a certain number of steps is reached. For the purpose of better understanding, each of the three stages is presented in sequence.

### 3.1. First Stage: Minimization of the Fidelity Term with SART

The first step is to solve the optimization problem described by Equation (13). A popular solution was proposed by Ge and Ming [33], and it can be computationally expressed by the iterative equation

(19) $$\begin{array}{c} {\tilde{\mu }}_{j}^{k}={\tilde{\mu }}_{j}^{k-1}+\lambda^{k}\frac{1}{a_{+j}}\sum_{i=1}^{N_{I}}\frac{a_{i,j}}{a_{+i}}\left({\hat{p}}_{i}-A_{i}\mu^{k-1}\right), \end{array}$$

where $a_{+j}=\sum_{i=1}^{N_{I}}a_{ij}>0$, $a_{+i}=\sum_{j=1}^{N_{J}}a_{ij}>0$, $A_{i}$ is the *i*-th line of A, *k* is the iteration index, and $0<\lambda^{k}<2$ is an arbitrary relaxation parameter [15,41]. To simplify the notation, one can establish $\Lambda^{+N_{J}}\in R^{N_{J}}\times R^{N_{J}}$ as a diagonal matrix with $\Lambda_{jj}^{+N_{J}}=\frac{1}{a_{+j}}$, and $\Lambda^{+N_{I}}\in R^{N_{I}}\times R^{N_{I}}$ also as a diagonal matrix with $\Lambda_{ii}^{+N_{I}}=\frac{1}{a_{+i}}$. Then, Equation (19) can be rewritten as

(20) $$\begin{array}{c} {\tilde{\mu }}^{k}={\tilde{\mu }}^{k-1}+\lambda^{k}\Lambda^{+N_{J}}A_{\Lambda }^{T}\Lambda^{N_{I}+}\left(p_{\Lambda }-A_{\Lambda }{\tilde{\mu }}^{k-1}\right), \end{array}$$

where the term $\lambda^{k}$ is usually constant and equal to 1. The method described in Equation (19) is commonly known as SART. This method produces a relatively noisy reconstruction, as can be observed in Section 4. We reinforce here that $\tilde{\mu }$ is the input of the second stage in the reconstruction process.

### 3.2. Second Stage: Bilateral Edge-Preserving with a Gradient Descent Method

In the second stage, the goal is to solve the optimization problem defined by

(21) $$\begin{array}{c} \hat{\mu }=\underset{\mu }{argmin}\left\{\tilde{\mu }-\gamma R_{BEP}\left(\tilde{\mu }\right)\right\}, \end{array}$$

where γ is a parameter that weights the contribution of the constraint $R_{BEP}$ (Equation (17)). The gradient descent method can be applied to solve this problem as

(22) $$\begin{array}{c} \mu^{k}=\mu^{k-1}-\gamma ▽R_{BEP}\left(\mu^{k-1}\right), \end{array}$$

resulting in an optimization problem written as follows (see Appendix A for details):

(23) $$\begin{array}{c} \hat{\mu }=\underset{\mu }{argmin}\left\{\rho_{a}\left(\tilde{\mu }\right)+\underset{l+m\geq 0}{\underset{︸}{\sum_{l=-q}^{q}\sum_{m=0}^{q}}}\alpha^{\left|l|+|m\right|}\rho_{c}\left(\tilde{\mu }-S_{x}^{l}S_{y}^{m}\tilde{\mu }\right)\right\}, \end{array}$$

where $\tilde{\mu }$, as defined in Equation (13), is the result of first-stage minimization; $\rho_{a}=\rho \left(s,a\right)$ is as in Equation (15) but with $s=\tilde{\mu }$; and $\rho_{c}=\rho \left(s,c\right)$ is the same as in Equation (15) but with a constant *c* instead of a constant *a* and $s=\tilde{\mu }-S_{x}^{l}S_{y}^{m}\tilde{\mu }$, where $S_{x}^{l}$ and $S_{y}^{m}$ are the same as in Equation (14). A computable matrix form was derived from Equation (23), and the result is shown below as

(24) $$\begin{array}{c} {\hat{\mu }}^{k}={\tilde{\mu }}^{k}-\gamma_{k}\left(H_{a}\left({\tilde{\mu }}^{k}\right)⊙{\tilde{\mu }}^{k}+\varphi \underset{l+m\geq 0}{\underset{︸}{\sum_{l=-q}^{q}\sum_{m=0}^{q}}}\alpha^{\left|l|+|m\right|}\left[I-S_{x}^{l}S_{y}^{m}\right]⊙H_{c}\left(M\right)⊙M\right), \end{array}$$

where $\gamma_{k}$ is an adjustment parameter to balance the *k*-th value of ${\tilde{\mu }}^{k}$ with the gradient descent contribution, $▽R_{BEP}\left({\tilde{\mu }}^{k-1}\right)$; φ is also an adjustment parameter, but it balances terms inside gradient descent; ⊙ is the element-by-element product of two matrices of compatible dimensions; and *I* is the identity matrix. The matrix $M={\tilde{\mu }}^{k}-S_{x}^{l}S_{y}^{m}{\tilde{\mu }}^{k}$ is the difference between ${\tilde{\mu }}^{k}$ and its version shifted by $S_{x}^{l}S_{y}^{m}$, and the operators $H_{a}\left(.\right)$ and $H_{c}\left(.\right)$ are defined, respectively, as

(25) $$H_{a}\left(x\right)=\frac{a}{\sqrt{a^{2}+x^{2}}},H_{c}\left(x\right)=\frac{c}{\sqrt{c^{2}+x^{2}}}.$$

$H_{a}\left({\tilde{\mu }}^{k}\right)⊙{\tilde{\mu }}^{k}$ and $H_{c}\left(M\right)⊙M$ are influence functions (as defined in Equation (16)) resulting from the application of the gradient descent method.

It is important to clarify that in Equation (22), μ appears with the upper index k-1 instead of *k* because the previous result of the gradient descent, $\mu^{k-1}$, feeds the calculation of the current value, $\mu^{k}$, and this is the manner in which gradient descent works. In contrast, Equation (24) shows $\tilde{\mu }$ with upper index *k* (as in $\hat{\mu }$) rather than k-1 because $\tilde{\mu }$ is obtained in the same interaction step, *k*, as $\hat{\mu }$, but in a previous stage denoted by the upper mark “tilde” $\left(\tilde{.}\right)$, while the current stage is denoted by the upper mark “hat” $\left(\hat{.}\right)$.

### 3.3. Third Stage: TV Minimization by Soft-threshold Filtering

The third stage (TV optimization, $R_{DGT}$) is to solve the problem ν=Dμ, where *D* is not invertible, as proposed by Yu and Wang [15] and shown below:

(26) $$\mu_{m,n}^{k}=\frac{1}{4}\left(2\mu_{m,n}^{k,a}+\mu_{m,n}^{k,b}+\mu_{m,n}^{k,c}\right),$$

with

(27) $$\begin{array}{c} \mu_{m,n}^{k,a}=\left\{\begin{array}{cc} \frac{2{\tilde{\mu }}_{m,n}^{k}+{\tilde{\mu }}_{m+1,n}^{k}+{\tilde{\mu }}_{m,n+1}^{k}}{4} & ,D_{m,n}{\tilde{\mu }}^{k}<\omega \\ {\tilde{\mu }}_{m,n}^{k}-\frac{\omega \left(2{\tilde{\mu }}_{m,n}^{k}-{\tilde{\mu }}_{m+1,n}^{k}-{\tilde{\mu }}_{m,n+1}^{k}\right)}{4D_{m,n}{\tilde{\mu }}^{k}} & ,D_{m,n}{\tilde{\mu }}^{k}\geq \omega , \end{array}\right. \end{array}$$

(28) $$\begin{array}{c} \mu_{m,n}^{k,b}=\left\{\begin{array}{cc} \frac{{\tilde{\mu }}_{m,n}^{k}+{\tilde{\mu }}_{m-1,n}^{k}}{2} & ,D_{m-1,n}{\tilde{\mu }}^{k}<\omega \\ {\tilde{\mu }}_{m,n}^{k}-\frac{\omega \left({\tilde{\mu }}_{m,n}^{k}-{\tilde{\mu }}_{m-1,n}^{k}\right)}{2D_{m-1,n}{\tilde{\mu }}^{k}} & ,D_{m-1,n}{\tilde{\mu }}^{k}\geq \omega , \end{array}\right. \end{array}$$

(29) $$\begin{array}{c} \mu_{m,n}^{k,c}=\left\{\begin{array}{cc} \frac{{\tilde{\mu }}_{m,n}^{k}+{\tilde{\mu }}_{m,n-1}^{k}}{2} & ,D_{m,n-1}{\tilde{\mu }}^{k}<\omega \\ {\tilde{\mu }}_{m,n}^{k}-\frac{\omega \left({\tilde{\mu }}_{m,n}^{k}-{\tilde{\mu }}_{m,n-1}^{k}\right)}{2D_{m,n-1}{\tilde{\mu }}^{k}} & ,D_{m,n-1}{\tilde{\mu }}^{k}\geq \omega , \end{array}\right. \end{array}$$

where ω is a pre-established threshold; ${\tilde{\mu }}^{k}={\left[{\tilde{\mu }}^{k}\right]}_{mn}$, with m=1,2,…,H and n=1,2,…,W, with *W* and *H* being the width and height of the reconstructed image, respectively. $D_{m,n}{\tilde{\mu }}^{k}$ is the DGT matrix as defined in Equation (7).

As explained in detail by [15] and observing Equation (27), when $D_{m,n}{\tilde{\mu }}^{k}<\omega$, the values of ${\tilde{\mu }}_{m,n}^{k}$, ${\tilde{\mu }}_{m+1,n}^{k}$, and ${\tilde{\mu }}_{m,n+1}^{k}$ must be adjusted so that $D_{m,n}{\tilde{\mu }}^{k}=0$. This means that if neighboring pixels in the reconstructed image are very close in value, it is likely that they have equal (or very close) values in the real image. Then, the method smooths the region around the pixel so that they look alike. Alternately, when $D_{m,n}{\tilde{\mu }}^{k}\geq \omega$, the goal is to reduce ${\left({\tilde{\mu }}_{m,n}^{k}-{\tilde{\mu }}_{m+1,n}^{k}\right)}^{2}$ and ${\left({\tilde{\mu }}_{m,n}^{k}-{\tilde{\mu }}_{m,n+1}^{k}\right)}^{2}$ but not cancel them. In this case, the method recognizes the differences between values of neighboring pixels as too significant to be totally eliminated. Instead, the differences are just softened.

### 3.4. Convergence and Convexity Considerations

**Figure 3:**
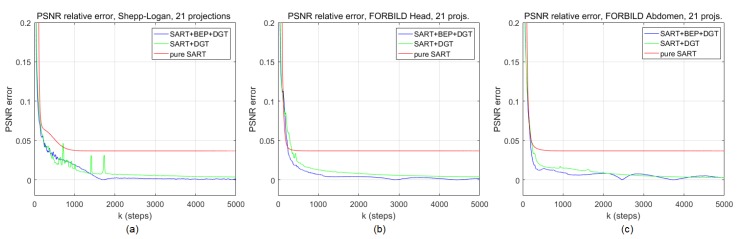
Peak signal-to-noise ratio (PSNR) difference along *k* iterations, 1<k≤5000, for the pure simultaneous algebraic reconstruction technique (SART), SART+discrete gradient transform (DGT), and SART+bilateral edge-preserving (BEP)+DGT reconstructions for (**a**) Shepp–Logan head phantom, (**b**) FORBILD head phantom, and (**c**) FORBILD abdomen phantom.

**Figure 4:**
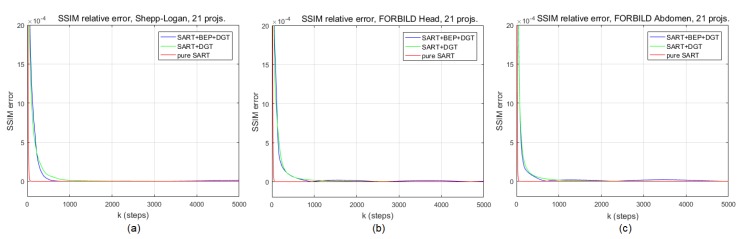
Sructural similarity (SSIM) difference along *k* iterations, 1<k≤5000, for pure SART, SART+DGT, and SART+BEP+DGT reconstructions for (**a**) Shepp–Logan head phantom, (**b**) FORBILD head phantom, and (**c**) FORBILD abdomen phantom.

**Figure 5:**
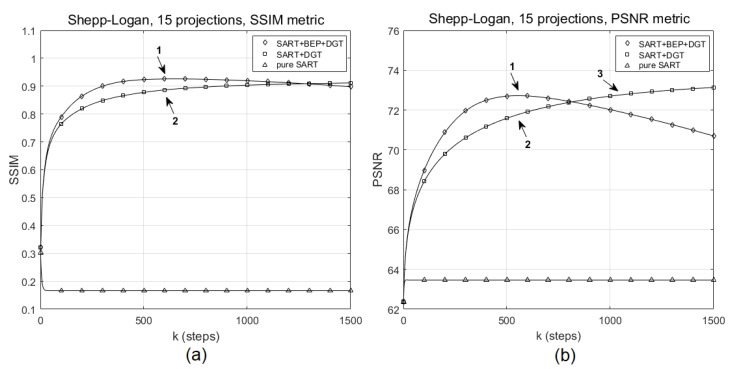
Evolution of (**a**) SSIM and (**b**) PSNR values for the reconstruction of the Shepp–Logan phantom with 15 projections for pure SART, SART+DGT, and SART+BEP+DGT.

**Figure 6:**
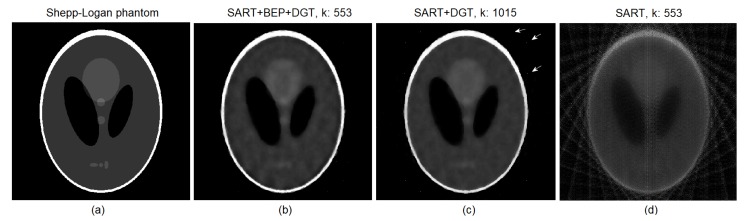
(**a**) The original Shepp–Logan head phantom and particular reconstructions for 15 projections (**b**) from SART+BEP+DGT with k=553 steps, PSNR: 72.7103, SSIM: 0.9240, (**c**) from SART+DGT with k=1015 steps, PSNR: 72.7103, SSIM: 0.8920, and (**d**) from pure SART with k=553 steps, PSNR: 63.4625, SSIM: 0.1662. All with SNR = 60 dB.

The model proposed in this work, represented by Equation 18, gives an important initial gain in terms of PSNR to the reconstruction of CT images, as reported in Section 4. However, it is important to know how this model behaves in long-term processing. It is therefore necessary to investigate its convergence. We do this empirically by comparing the PSNR values of the cost function between the iterations *k* and k-1, with 1<k≤5000. We present in Figure 3 a chart for each image used in the simulations of Section 4, and more specifically for the graphs and images of Figures 5 and 6 (Shepp–Logan head phantom), 7 and 8 (FORBILD head phantom), 9 and 10 (FORBILD abdomen phantom). It is worth noting that in all cases shown in Figure 3, the SART+BEP+DGT method is consistent with respect to convergence and presents better error reduction in terms of the PSNR metric. This simulation uses the same initial values defined in Section 4. The same situation applies when we graphically analyze the convergence of the proposed method using the SSIM metric, as can be seen in Figure 4.

It is noteworthy that, according to Charbonnier et al. [24], the function described in Equation (15) is convex, and therefore, it would be possible to apply an iTV-style minimization procedure [42] to assure data consistency and regularization term improvement in each iteration step.

## 4. Experiments and Results

In the experiments, we used the synthetic images presented in Section 1.2, that is, Shepp–Logan head phantom, FORBILD head phantom, and FORBILD abdomen phantom. The signal from the CT equipment is simulated according to the model in Equation (1) addressing the low dosage scenario, considering a limited number of projections (meaning a limited number of scanning angles). On the image reconstruction side, we use the model y=Ax+e, which, as discussed in Section 1.1, denotes an inverse and ill-posed problem, where A ($N_{I}\times N_{J}$) is the matrix that describes the capture system, *x* ($N_{J}\times 1$) is the phantom represented lexicographically, and *e* ($N_{J}\times 1$) is the error, whose features were presented in Section 1.2. It is worth remembering that *x* is the image we intend to reconstruct from the noise signal *y* and, in the modeling process presented in Section 2 and Section 3, we use the variable μ to represent it. By improving the system description, $N_{I}=n_{l}n_{\theta }$ is the number of projections, where $n_{l}$ is the number of projection lines (i.e., the number of detectors) for each scan angle, and $n_{\theta }$ is the total number of scan angles. $n_{\theta }$ is the parameter whose value should be changed when the intention is to set a new dosage value, i.e., when we want to define a different (lower) number of projections, $N_{I}$. The image has dimensions d×d, where $d=\sqrt{N_{J}}=2^{R}$, $R\in N_{+}$ (positive natural). In this work, we use R=9(d=512), and therefore, $N_{J}\left(=d^{2}\right)=262,144$, and $N_{I}\left(=n_{l}n_{\theta }\right)=300n_{\theta }$, with $n_{l}=300$ detectors. Thus, A has dimensions $300n_{\theta }\times 262,144$, which are compatible with the dimensions of *y* and μ, respectively, i.e., $300n_{\theta }\times 1$ and 262,144×1. It is important to note that the $N_{I}$ dimension of *A* (and *y*) is related to the number of scan angles, $n_{\theta }$, and this number of angles is what determines if the signal is of low (or regular) dosage, as discussed in Section 1, according to the ALARA principle. For a low dosage, we consider subsets of Θ, i.e., equally spaced sets of integer values between 0 and 179 degrees named $\Theta_{g}$. For example, $\Theta_{g=5}=\left\{0,44,88,132,176\right\}$ would be a possible subset, in which the g=5 angles are equally spaced at 44 degrees. Using this notation, Θ is equivalent to $\Theta_{180}$, meaning that there are g=180 scan angles equally spaced by 1 degree (which represents a regular dosage). In the experiments with low dosage, the sets $\Theta_{g}$, with *g* in {15,30}, will be used. A was obtained for a parallel architecture scanner.

By observing the objective function optimization process detailed in Section 3, and in alignment with the proposal in Section 1.3, we design a test framework that involves (1) the execution of the first stage (Section 3.1) alternating with the third stage (Section 3.3), which we will call here SART+DGT, and (2) execution of the first, second (Section 3.2), and third stages alternately and in this sequence, named SART+BEP+DGT. Simulations are shown in Table 1.

For both arrangements (SART+BEP and SART+BEP+DGT), the first stage is mandatory because it is the core of the reconstruction process. That is, it represents the optimization of the fidelity term in Equation (13). Because of its omnipresence, we also show simulations with SART only, which serve as a basis for comparing how much constraint terms actually contribute to the reconstruction process. The subsequent stages represent the application of constraint terms as described in Section 3.2 and Section 3.3, respectively, Equations (24) and (26). For each of these test arrangements, it was arbitrarily established that the iterator, *k*, ranges from 1 to *L*, with 350≤L<1500, approximately. Because Gaussian noise and the Poisson process are random, each experiment is performed a considerable number of times, defined arbitrarily as 101 executions by experiment, and each result in Table 1 is the mean of the 101 SSIM and PSNR values. It is important to note that the result presented for each experiment (with a particular additive Gaussian noise or a certain number of projections) is the mean of 101 executions performed. Each execution produces a particular SSIM and PSNR result. We do not average pixels in any reconstructed image, but rather the SSIM and PSNR of the 101 executions performed for each testing case. The idea of using the average of a considerable number of iterations is based on the central limit theorem, which states that the arithmetic mean of a sufficiently large number of iterates of independent random variables will be approximately normally distributed, regardless of the underlying distribution, provided that each iteration has a finite expected value.

### Low Dosage Tests and Results

As recommended by the ALARA principle, an alternative to reduce the total amount of radiation applied to a patient is decreasing the number of projections in the acquisition of the CT signal. According to the signal model proposed in Equation (1), we will consider the projections as individually influenced by Gaussian additive noise, and the low dosage signal is provided by reducing the number of scanning angles. In the batch of tests with low dosage projections, we consider using the sets of angles $\Theta_{g}$, with *g* in {15,30}, where *g* is the amount of angles in $\Theta_{g}$ (remember that in a regular dosage, we have 180 angles, from $0^{o},\ldots ,179^{o}$). In our model, these values represent a reduced amount of photon emission, which can be understood as a low radiation dosage. All low dosage presented in this section is performed with signal-to-noise ratio (SNR) =32, 46, and 60 dB. The SART stage used λ=1, according [15] and [41]. The DGT stage maintained β=1, as discussed in Section 2.1, and used as a threshold, ω, the average of the DGT for each *k* iteration. The BEP stage used γ=0.001, φ=0.150 (Section 3.2), a=0.5, q=3, α=0.6, and c=0.1 (Section 2.2). All parameters were empirically set.

A batch of experiments using the set $\Theta_{g}$, with *g* in {15,30}, of projections for SART+BEP+DGT (named here as method A), SART+DGT (named here as B), and pure SART (named here as method C) methods are shown in Table 1 for PSNR and SSIM metrics, using the FORBILD abdomen phantom (FA), FORBILD head phantom (FH), and Shepp–Logan head phantom (SL) synthetic images. Analyzing the results for the PSNR metric with 15 and 30 projections, it is observed that for k=350 steps, the results of the proposed method present a higher PSNR value in general. The exceptions are the FA and FH images, with an SNR of 32 dB. However, for k=700 and 1000 steps and 30 projections, the results favor the SART+DGT method according to the PSNR metric. For 15 projections, results for k=700 steps favor the proposed method in most tests performed with PSNR metrics. For the SSIM metric, the proposed method presents interesting results when compared to the SART+DGT method.

For the reconstruction of Shepp–Logan head phantom with 15 angles of projection, Figure 5 shows the evolution of the SSIM and PSNR values for SART+BEP+DGT (proposed), SART+DGT, and pure SART methods for SNR = 60 dB. In this particular experiment, marker 1 in Figure 5a indicates the highest SSIM value, 0.9240, reached by the proposed method and corresponding to the highest PSNR value, 72.7103, indicated by marker 1 in Figure 5b. Marker 2 shows in Figure 5a,b, respectively, the SSIM (0.8819 ) and PSNR ( 71.7521 ) values obtained in step k=551. Marker 3 in Figure 5b highlights the point at which the SART+DGT method reaches the same PSNR value as the proposed method, in step k=1015, approximately, and the graphs in Figure 5 agree with Table 1.

Looking closely at Figure 6b,c, it is possible to note the presence of random noise (indicated by the white arrows) that manifests as small white dots in Figure 6c, while in Figure 6b this phenomenon is not easily perceived. This is because the BEP regularization used in the proposed method (Figure 6b) tends to eliminate noise faster. However, the reconstruction performed by the SART+DGT method produces a more homogeneous image, as shown in Figure 6c. This is also related to the elimination of random noise by the introduction of BEP regularization in the reconstruction process. Figure 6d presents the result of the image reconstruction using the pure SART method.

**Figure 7:**
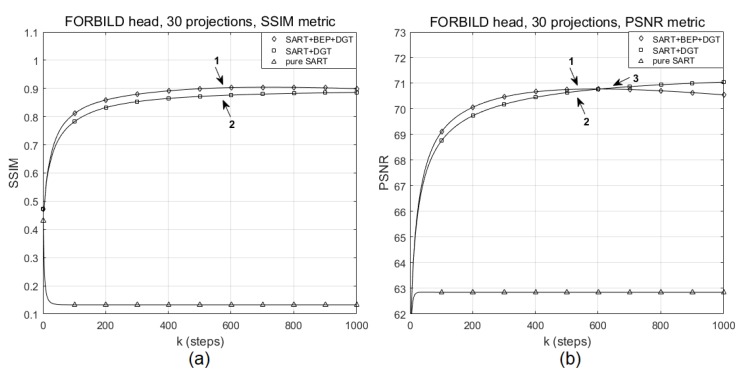
Evolution of (**a**) SSIM and (**b**) PSNR values for a particular reconstruction of the FORBILD head phantom with 30 projections using the pure SART, SART+DGT, and SART+BEP+DGT methods.

**Figure 8:**
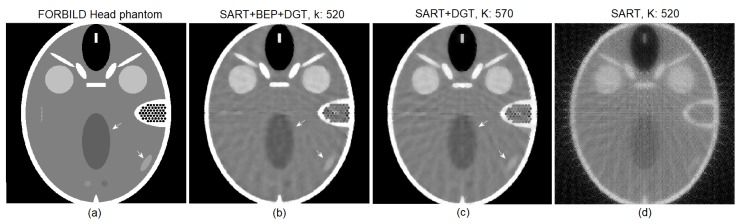
(**a**) The original FORBILD head phantom and particular reconstructions for 30 projections (**b**) from SART+BEP+DGT with k=520 steps, PSNR: 70.7700, SSIM: 0.9015, (**c**) from SART+DGT with k=570 steps, PSNR: 70.7706, SSIM: 0.8774, and (**d**) from pure SART with k=520 steps, PSNR: 62.8476, SSIM: 0.1332. All with SNR = 46 dB.

For the reconstruction of the FORBILD head phantom with 30 projections with SNR = 46 dB, shown in Figure 7, we observe that the best PSNR (70.7700) obtained by the proposed method in step k=520 (Figure 7b, marker 1) is reached by the SART+DGT method in step k=570 (marker 3). Marker 2 shows, in Figure 7a,b, respectively, the SSIM (0.8728) and PSNR (70.6901) values obtained in step k=685. The SSIM values remain higher for the proposed method, according the graph of Figure 7a. We show the evolution of the pure SART method in terms of SSIM and PSNR for comparison purposes only. Observing the reconstructions shown in Figure 8b (with k=520 steps, PSNR =70.7700, SSIM =0.9015) and Figure 8c (with k=570 steps, PSNR =70.7706, SSIM =0.8774), the results are low in quality due to the number of projections, and the images practically do not present a difference, except for a better contrast level presented by Figure 8b. The pure SART reconstruction is shown in Figure 8d.

**Figure 9:**
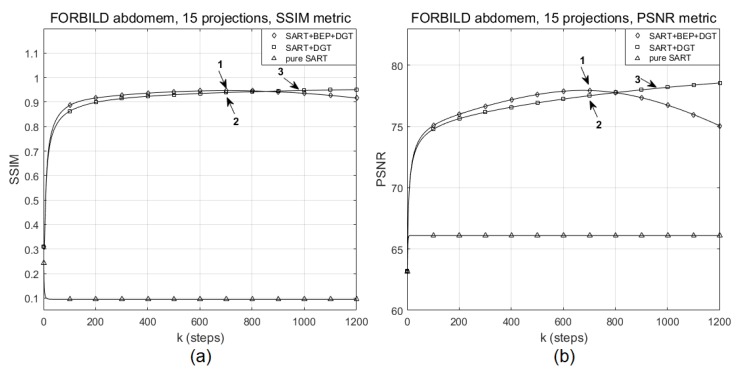
Evolution of (**a**) SSIM and (**b**) PSNR values for a particular reconstruction of the FORBILD abdomen phantom with 15 projections for pure SART, SART+DGT, and SART+BEP+DGT methods.

**Figure 10:**
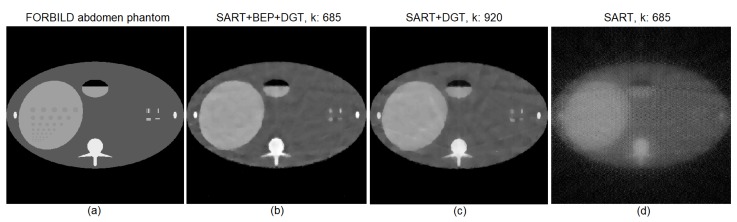
(**a**) The original FORBILD abdomen phantom and particular reconstructions for 15 projections (**b**) from SART+BEP+DGT with k=685 steps, PSNR: 77.7180, SSIM: 0.9419, (**c**) from SART+DGT with k=920 steps, PSNR: 77.7197, SSIM: 0.9397, and (**d**) pure SART with k=685 steps, PSNR: 66.0972, SSIM: 0.0946.

Figure 9 shows the evolution of the SSIM and PSNR values for the reconstruction with 15 angles of projection with SNR = 60 dB for the FORBILD abdomen phantom using the SART+BEP+DGT, SART+DGT, and pure SART methods. In this particular experiment, marker 1 in Figure 9a indicates the highest SSIM value, 0.9419, reached by the proposed method and corresponding to the highest PSNR value, 77.7180, indicated by marker 1 in Figure 9b, both obtained in step k=685. Marker 2 shows, in Figure 9a,b, respectively, the SSIM (0.9327) and PSNR (77.2279) values obtained in step k=685. Marker 3 in Figure 9b highlights the point at which the SART+DGT method reaches the same PSNR value of the proposed method, in step k=920, approximately. The image of Figure 10b shows a tendency to eliminate the characteristic lines and bands of the reconstruction process with few scanning angles.

**Figure 11:**
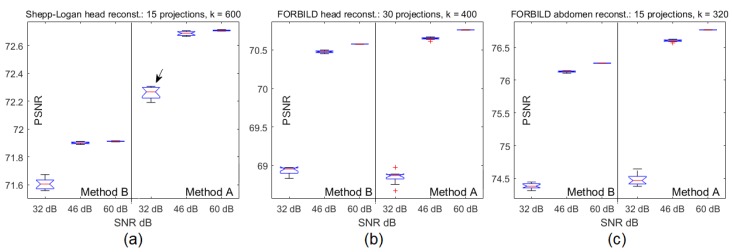
(**a**) Box plot graph for the Shepp–Logan head reconstruction with 15 projections and k=600, (**b**) box plot graph for the FORBILD head phantom with 30 projections and k=400, and (**c**) box plot graph for the FORBILD abdomen phantom with 15 projections and k=320.

Each of the examples of reconstructed images shown in Figure 6, Figure 8 and Figure 10, and their SSIM and PSNR graphs in Figure 5, Figure 7, and Figure 9, respectively, result from a single execution test pinched from a set of 101 executions. Figure 11 shows box plot graphs for various situations, combining image to be reconstructed, SNR dB value, number of projections, method used, and step *k* of the execution. As an example, for the reconstruction of Shepp–Logan with 15 projections, method *A* and SNR = 32 dB using the column (step) k=600 of the processing matrix 101×1000 is highlighted in the graph of Figure 11a. Table 2 shows the details of each element of the box plot graphs in Figure 11, and it is straightforward to note that the standard deviation (Table 2) increases with noise (Figure 11).

## 5. Conclusions and Final Comments

The proposed method, composed by the steps (1) SART reconstruction, (2) BEP adaptive minimization, and (3) TV minimization via DGT, synthesized in Equation (18), presents, in the first steps of the processing, a more pronounced reduction in the noise level of the reconstructed image both for SSIM and PSNR metrics, as can be seen in Section 4. It is important to emphasize that the tests were done with 15 and 30 projections, as shown in Table 1. At some point, the proposed method reaches its maximum PSNR value. It can be observed that at this point (maximum PSNR), the images are reasonably intelligible. From this point forward, the SART+DGT method gives higher values of PSNR and, consequently, a less noisy reconstruction. Even after the apex of the proposed method with regard to the value of PSNR, the value of SSIM remains above in many of the cases studied, when compared to the result of the SART+DGT method. The best values for SSIM generally result in images with better contrast, and this is very important for artifact viewing and contour distinction in the reconstructed image. Structural similarity works considering morphological features in the evaluation of reconstruction results and for this reason presents results more suitable to human standards, when compared with the PSNR metric. However, the main disadvantage of this method is that in a practical application, we cannot know the maximum PSNR since we do not have an original image for comparison. On the other hand, the advantage of the proposed method is that it delivers results earlier in the reconstruction process.

The use of BEP minimization soon after the SART reconstruction, as explained in Section 3, is intended to promote image noise reduction in the reconstruction process, delivering a less noisy image to the later stage (of minimization by TV using DGT). In fact, the noise reduction occurs up to a certain point, and although it is not possible in a practical application to define the ideal stopping point (maximum PSNR), it may be possible to estimate this point based on the type of image and the number of projections used.

## Figures and Tables

**Table 1:** Comparison of computer tomography (CT) reconstruction methods A (SART+BEP+DGT), B (SART+DGT), and C (pure SART) for the Shepp–Logan (SL), FORBILD abdomen (FA), and FORBILD head (FH) phantom images using PSNR and SSIM metrics for 15 and 30 projections and signal-to-noise ratio (SNR) of 32, 46, and 60 dB. Each result is the mean of 101 executions of a particular testing case. The values in bold represent the highest value comparing methods A, B and C for each metric (PSNR or SSIM), number of projections (15 or 30) and iterations (350, 700 or 1000).

			PSNR Metric						SSIM Metric					
Image	Noise (dB)	Method	30 projections			15 Projections			30 Projections			15 Projections		
			k = 350	700	1000	k = 350	700	1000	k = 350	700	1000	k = 350	700	1000
		A	75.9859	75.8311	75.5142	**74.4987**	74.502	73.8998	**0.8522**	**0.8568**	**0.8548**	**0.8424**	**0.8506**	**0.8480**
	32	B	**76.1405**	**76.2478**	**76.2701**	74.4034	**74.5392**	**74.587**	0.8389	0.8452	0.8470	0.8277	0.8368	0.8403
		C	67.9001	67.9001	67.9001	66.0238	66.0238	66.0238	0.1281	0.1281	0.1281	0.0905	0.0905	0.0905
		A	**79.7397**	79.7947	79.1235	**76.7531**	**77.7001**	76.634	**0.9589**	0.9625	0.9572	**0.9278**	**0.9418**	0.9318
FA	46	B	79.5257	**80.759**	**81.2113**	76.2466	77.2650	**77.8593**	0.9506	**0.9630**	**0.9662**	0.9147	0.9334	**0.9412**
		C	68.1199	68.1199	68.1199	66.0941	66.0941	66.0941	0.1418	0.1418	0.1418	0.0942	0.0942	0.0942
		A	**80.0246**	80.0840	79.3894	**76.9335**	**77.951**	76.7656	**0.9634**	0.9665	0.9615	**0.9328**	**0.9468**	0.9356
	60	B	79.7982	**81.2432**	**81.8295**	76.3869	77.5277	**78.2139**	0.9564	**0.9692**	**0.9727**	0.9194	0.9390	**0.9473**
		C	68.1290	68.1290	68.1290	66.0973	66.0973	66.0973	0.1424	0.1424	0.1424	0.0947	0.0947	0.0947
		A	68.8491	68.7420	68.5457	**67.4093**	67.0085	66.2663	**0.753**	**0.7624**	**0.7607**	**0.7292**	**0.7313**	**0.7203**
	32	B	68.8981	**69.0161**	**69.043**	67.3406	**67.4181**	**67.4202**	0.7236	0.7340	0.7368	0.6998	0.7112	0.7143
		C	62.6576	62.6576	62.6576	61.3241	61.3241	61.3241	0.1096	0.1096	0.1096	0.1013	0.1013	0.1013
		A	**70.5681**	70.7268	70.5159	**68.3913**	**68.4761**	68.1521	**0.8852**	**0.9028**	**0.898**	**0.8288**	**0.8394**	**0.8361**
FH	46	B	70.3511	**70.9038**	**71.0856**	68.0724	68.4681	**68.6376**	0.8605	0.8815	0.8876	0.7985	0.8216	0.8292
		C	62.8480	62.8480	62.8480	61.3975	61.3975	61.3975	0.1327	0.1327	0.1327	0.1130	0.1130	0.1130
		A	**70.6733**	70.8539	70.6321	**68.4433**	**68.5563**	68.2407	**0.8919**	**0.9100**	**0.9049**	**0.8337**	**0.8450**	**0.8420**
	60	B	70.4422	**71.0466**	**71.2518**	68.1110	68.5327	**68.7161**	0.8679	0.8899	0.8964	0.8033	0.8274	0.8356
		C	62.8551	62.8551	62.8551	61.4002	61.4002	61.4002	0.1342	0.1342	0.1342	0.1136	0.1136	0.1136
		A	**74.0945**	74.0583	73.8128	**71.9549**	**72.1447**	71.5547	**0.9325**	**0.9358**	**0.9350**	**0.8938**	**0.9066**	**0.8989**
	32	B	73.5586	**74.3921**	**74.6588**	70.7452	71.812	**72.2027**	0.9036	0.9151	0.9174	0.8433	0.8740	0.8831
		C	64.5268	64.5268	64.5268	63.4489	63.4489	63.4489	0.1800	0.1800	0.1800	0.1604	0.1604	0.1604
		A	**74.6383**	74.6475	74.3422	**72.2586**	**72.5832**	72.0034	**0.9542**	**0.9579**	**0.9572**	**0.9093**	**0.9251**	**0.9186**
SL	46	B	73.9486	**75.1170**	**75.5809**	70.8990	72.1570	**72.6781**	0.9298	0.9444	0.9477	0.8575	0.8917	0.9026
		C	64.5592	64.5592	64.5592	63.4619	63.4619	63.4619	0.1940	0.1940	0.1940	0.1661	0.1661	0.1661
		A	**74.6588**	74.6647	74.3552	**72.2741**	**72.6033**	72.0213	**0.955**	**0.9587**	**0.9581**	**0.9099**	**0.9257**	**0.9193**
	60	B	73.9636	**75.1504**	**75.6263**	70.9047	72.1699	**72.6975**	0.9309	0.9458	0.9491	0.8580	0.8925	0.9036
		C	64.5606	64.5606	64.5606	63.4625	63.4625	63.4625	0.1946	0.1946	0.1946	0.1663	0.1663	0.1663

**Table 2:** Mean, median, maximum, minimum, standard deviation, and number of outliers of box plot element in Figure 11a.

Image	Method	SNR dB	Mean	Median	Max	Min	Standard Deviation
		32	71.60589	71.60400	71.67289	71.55672	0.03613
	B	46	71.90013	71.89770	71.91010	71.88857	0.00725
SL		60	71.91110	71.91103	71.91474	71.90779	0.00199
		32	72.25797	72.26654	72.30637	72.19037	0.04360
	A	46	72.68626	72.68664	72.70573	72.66494	0.01435
		60	72.70719	72.70709	72.71359	72.70282	0.00356

## Abbreviations

The following abbreviations are used in this manuscript:

ALARA	As-low-as-reasonably-achievable
AMP	Approximate message passing
ART	Algebraic reconstruction technique
BEP	Bilateral edge preserving
CT	Computer tomography
DCT-GAMP	Denoising CT generalized Approximate message passing
DGT	Discrete gradient transform
FBP	Filtered backprojection
FFT	Fast Fourier transform
MAP	Maximum a posteriori
MMSE	Minimum mean square error
OS-SART	Ordered subset simultaneous algebraic reconstruction technique
PSNR	Peak signal-to-noise ratio
TV	Total variation
SART	Simultaneous algebraic reconstruction technique
SSIM]	Structural similarity
VW-SART	Variable weighted simultaneous algebraic reconstruction technique

## Appendix A

With Equation (22) in mind and substituting Equation (15) into the core (within the braces) of Equation (23), we have

(A1) $$▽R_{BEP}\left(\tilde{\mu }\right)=\frac{\partial }{\partial \tilde{\mu }}\left(a\sqrt{a^{2}+{\tilde{\mu }}^{2}}+\varphi \underset{l+m\geq 0}{\underset{︸}{\sum_{l=-q}^{q}\sum_{m=0}^{q}}}\alpha^{\left|l|+|m\right|}c\sqrt{c^{2}+{\left(\tilde{\mu }-S_{x}^{l}S_{y}^{m}\tilde{\mu }\right)}^{2}}\right),$$

and performing the derivative in relation to $\tilde{\mu }$ results in

(A2) $$▽R_{BEP}\left(\tilde{\mu }\right)=\frac{a\tilde{\mu }}{\sqrt{a^{2}+{\tilde{\mu }}^{2}}}+\varphi \underset{l+m\geq 0}{\underset{︸}{\sum_{l=-q}^{q}\sum_{m=0}^{q}}}\alpha^{\left|l|+|m\right|}\frac{c\left(I-S_{x}^{l}S_{y}^{m}\right)\left(\tilde{\mu }-\tilde{\mu }S_{x}^{l}S_{y}^{m}\right)}{\sqrt{c^{2}+{\left(\tilde{\mu }-S_{x}^{l}S_{y}^{m}\tilde{\mu }\right)}^{2}}}.$$

Assuming the same considerations and notation presented in Section 3.2, Equation (A1) can be rewritten as

(A3) $$▽R_{BEP}\left(\tilde{\mu }\right)=H_{a}\left(\tilde{\mu }\right)⊙\tilde{\mu }+\varphi \underset{l+m\geq 0}{\underset{︸}{\sum_{l=-q}^{q}\sum_{m=0}^{q}}}\alpha^{\left|l|+|m\right|}\left[I-S_{x}^{-l}S_{y}^{-m}\right]\left(M\right)H_{c}\left(M\right),$$

and Equation (23) can be written in compact form as

(A4) $$\begin{array}{c} {\hat{\mu }}^{k}={\tilde{\mu }}^{k}-\gamma ▽R_{BEP}\left({\tilde{\mu }}^{k}\right) \end{array}$$

and in its expanded form as

(A5) $$\begin{array}{c} {\hat{\mu }}^{k}={\tilde{\mu }}^{k}-\gamma_{k}\left(H_{a}\left({\tilde{\mu }}^{k}\right)⊙{\tilde{\mu }}^{k}+\varphi \underset{l+m\geq 0}{\underset{︸}{\sum_{l=-q}^{q}\sum_{m=0}^{q}}}\alpha^{\left|l|+|m\right|}\left[I-S_{x}^{l}S_{y}^{m}\right]⊙H_{c}\left(M\right)⊙M\right). \end{array}$$
